# Risk factors associated with short term mortality changes over time, after arrival to the emergency department

**DOI:** 10.1186/s13049-018-0493-2

**Published:** 2018-04-20

**Authors:** Camilla Nørgaard Bech, Mikkel Brabrand, Søren Mikkelsen, Annmarie Lassen

**Affiliations:** 1 Department of Emergency Medicine, Odense University Hospital, Sdr. Boulevard 29, entrance 130, 1st floor, DK-5000 Odense C, Denmark; 2 Department of Emergency Medicine, University of Southern Denmark, Odense University Hospital, Odense, Denmark; 3 Mobile Emergency Care Unit, Department of Anaesthesiology and Intensive Care Medicine, Odense University Hospital, Odense, Denmark; 4 Department of Emergency Medicine, Odense University Hospital, Odense, Denmark

**Keywords:** Emergency Department, Short term mortality, Risk factors, Observational cohort study

## Abstract

**Background:**

Preventing death is the most important outcome pursued in the Emergency Department. Prompt accurate assessment, followed by competent and efficient investigation and treatment is the recipe sought. Abnormal physiological measurements are common antecedents to deterioration and therefore a cornerstone in many risk stratification tools. Some risk factors have their impact during the first few days after admittance, others have higher impact on 30 day mortality. Understanding the variance in impact of risk factors is relevant for future composition of risk stratification models.

**Methods:**

We included patients aged 18 years or older, registered at the Emergency Department at Odense University Hospital from April 1st 2012 to September 30th 2014. We performed multivariate logistic regressions, adjusted for age, gender and comorbidity, to describe the relationship between potential risk factors and measures of short term mortality.

**Results:**

A total of 43,178 were eligible for analysis. Median age was 56 (IQR 36–72) and 48.3% were males. The over-all 30-day-mortality was 4%. One third of deaths occurred within the first 2 days.

Higher age, male gender and comorbidity are all associated with immediate, 0-2 day, 3-7 day and 8–30 day mortality. The degree of acuteness at arrival defined by urgency-level, physician-assisted transfer to the Emergency Department and abnormal vital parameters are associated with 0-2 day mortality. High temperature at arrival shows no association in either mortality-group. Missing values are associated with immediate and 0–2 day mortality, but no association with mortality after 7 days.

**Discussion:**

Abnormal vital parameters and degree of acuity at admission were strongly associated with mortality in the first hours and days after admission, where after the association decreased. The effect of other risk factors such as male gender, comorbidity and high age were time stable or even increasing over time..

**Conclusions:**

The over-all 30-day mortality was 4%. Physiology–related risk factors varied in strength of association throughout different mortality outcome measures.

**Electronic supplementary material:**

The online version of this article (10.1186/s13049-018-0493-2) contains supplementary material, which is available to authorized users.

## Background

Various early warning scores are used to identify patients, who are likely to deteriorate while hospitalized. Most of these warning scores are based on antecedents to deterioration such as abnormal vital values [1–6]. Other mortality-associated risk factors, such as lab results or time related factors are relevant in some – but not all settings, and the optimal method for risk assessment is often debated [7–17].

The impact on mortality of different risk factors might vary over time. Some risk factors are probably more important within the first few hours or days after arrival, while other risk factors have higher impact on 8–30 day mortality. Important risk factors related to death during the first few days, but not important for later death might be overlooked or blurred if timing of the outcome is not taken into account.

Since variation in impact of risk factor according to time after symptom occurrence (or arrival to the ED) is closely related to the efficacy of risk scores, our aim of the present study is to describe risk factors associated with mortality, distributed on four mortality-timespans after arrival to the ED; immediate death (0-4 h), 4 h-2 days, 3-7 days and 8–30 days.

## Methods

### Study design and setting

This observational cohort study collected data on adults, who arrived to the ED at Odense University Hospital (OUH) during a 2 ½ year period. OUH is a tertiary hospital which also serves as a primary hospital for a population of 288,000 citizens. The ED provides 24-h acute care with 105,000 visits per year (children and adults). The ED receives all acutely patients referred from either a primary care physician or patients who arrive by ambulance. The general admitting services for cardiology, hematology, oncology and obstetrics is placed elsewhere in-hospital.

The pre-hospital primary response is an ambulance manned with two emergency medical technicians or paramedics. The ambulance may be supplemented by a ground-based Mobile Emergency care Unit (MECU) manned with a specialist in anesthesiology [18]. In Denmark, hospital care is free of charge as part of the tax-funded healthcare system.

### Participants

This study included all patients above 17 years, who arrived alive to the ED between April 1st 2012 and September 30th 2014. Patients with minor orthopedic injuries and patients without a Danish personal identification number were excluded. In order to avoid bias from repeated measurements, only first time ED-visit in the study period and the first set of recorded vital parameters were included for analysis.

### Data sources and variables

The primary outcome was short term mortality, reported as immediate death (0–4 h after arrival), 4 h-2 day mortality, 3-7 day and 8–30 day mortality. Arrival day were defined as day 0.

Each patient-visit was linked to information from the two large population-based registries deriving from the unique Danish personal identification number: The Danish National Patient Registry and the Danish Civil Registration System [19, 20]. Collected data included patient demographics, with comorbidity defined by a condition recorded in a discharge diagnosis within 10 year prior to the index date, and described by Charlson Comorbidity Index [21]. Discharge diagnoses from the ED were collected and divided into major ICD-10 categories. We also included variables of day- or night-time and weekday or weekend. Mode of arrival was analyzed according to arrival at own accord, by ambulance or by ambulance assisted by MECU.

The first registered set of vital parameters; Glasgow coma score, heart rate, systolic blood pressure, temperature, peripheral oxygen saturation and respiratory rate were included. These data came primarily from electronic ED patient records, registered upon arrival to the ED. Secondly vital parameters from pre-hospital data registered in the MECU database or in ambulance records were used. Vital parameters were then divided into predefined strata, based on published guidelines [22].

The level of urgency was categorized according to the triage-assessment registered a arrival. The suspected degree of life or limb threat, assessment of vital parameters and the patient’s presenting complaint are part of our triage system [1, 2] (see supplemental 1 for details on vital values). We defined the highest urgency-level as “very urgent”, a patient-category with immediate assistance of a dedicated trauma- or medical emergency response team upon arrival to the ED. Our level 2 category of urgency was named “urgent” and was merged by the two middle triage-levels, 2 and 3, which consists of patients who needs to be seen by a physician within 15 or 60 min. (Additional file 1).

### Statistical methods

Demographic data are described as median, quartiles and range (where appropriate). Proportions are presented with 95% confidence intervals, based on binomial distribution. Patients were followed to death or 30 days following index date, whichever came first.

Logistic regressions determined the association between exposure-variables, distributed on four levels of short time mortality. Multivariate logistic regression, adjusted for age group, gender and comorbidity was used to determine odds ratios (OR).

Missing values were handled as independent variables throughout the analysis.

All statistical analyses were performed with STATA software (V. 14.0 Stata Corporation LP, Texas, USA).

## Results

A total of 182,032 contacts with an average of 199 contacts per day were initially registered. After excluding patients dead at arrival, and patients with minor orthopedic injuries, we included 43,178 individual adult patients with a first time contact to the ED. Median age was 56 (IQR 36–72) and 48.3% were male.

Approximately three quarters of the cohort arrived at their own accord. The majority of arrivals occurred during daytime in weekdays and was evaluated as “urgent” at first assessment in the ED. Vital parameters were in general within predefined “normal” reference limits, with a proportion of missing values ranging from 15.5% in heart rate to 30.0% in GCS (Table 1).

**Table 1:** Patient characteristics, time and urgency registrations

	n	%
Overall	43.178	100
Age, median (years (IQR))	56	(36–72)
Gender		
Female	22.318	51.7
Male	20.861	48.3
Charlson comorbidity Index		
0	25.962	60.1
1	7.121	16.5
≥ 2	10.095	23.4
Mode of arriving		
Own accord	32.171	74.5
Ambulance	10.208	23.6
MECU	799	1.9
Time of arrival		
8–15.59	21.461	49.7
16–23.59	15.262	35.4
00–6.59	6.455	14.9
Day of arrival		
Mon-Thursday	25.089	58.1
Fri-Sunday	18.089	41.9
Urgency		
Non-urgent	11.367	26.3
Urgent	25.063	58.0
Very urgent	1.833	4.3
missing	4.915	11.4
Vital parameters		
Peripheral oxygen saturation (%)		
Normal	33.359	77.3
< 90%	858	2.0
missing	8.961	20.7
Respiratory rate (rr/min)		
Normal	31.471	72.9
< 5	30	0.1
> 36	351	0.8
missing	11.326	26.2
Systolic Blood Pressure (mmHg)		
Normal	36.024	83.4
< 90	477	1.1
missing	6.677	15.5
Heart rate (hr/min)		
Normal	36,218	83.9
< 40	99	0.2
> 140	284	0.6
missing	6.587	15.3
Glascow Coma Score		
14–15	29.327	67.9
3–13	905	2.1
missing	12,946	30.0
Temperature		
Normal	31.817	73.7
< 35	89	0.2
> 39	631	1.5
missing	10,641	24.6

**Fig. 1:**
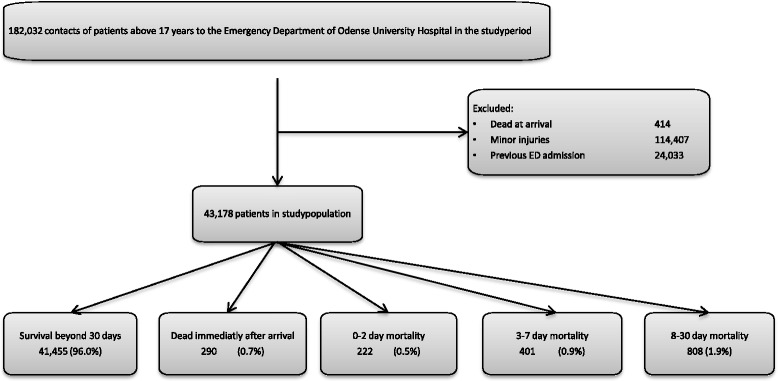
Flowchart describing selection of cohort and distribution of outcome

The overall 30 day mortality was 4%, with 0.7% dead within 4 h after arrival, 0.5% in 0–2 day mortality, 0.9% in 3–7 day mortality and 1.9% dead in 8–30 day mortality (Fig. 1). Conclusively, 30% of the patients who died within 30 days, died within the first 2 days after arrival to the ED.

Strong risk factors for immediate mortality were abnormal vital values as well as high urgency level at arrival. The strength of association with mortality of these risk factors declined considerable according to increasing time from arrival till death (Table 2).

**Table 2:** Odds ratios with 95% confidence intervals, distributed on 4 mortality levels

	Immediatly dead (0-4 h)						4 h-2 day mortality					3–7 day mortality					8–30 day mortality			
Gender		Crude OR	95% CI	Adj OR^a^	95% CI		Crude OR	95% CI	Adj OR^a^	95% CI		Crude OR	95% CI	Adj OR^a^	95% CI		Crude OR	95% CI	Adj OR^a^	95% CI
Female	134	1.0		1.0		154	1.0		1.0		187	1.0		1.0		393	1.0		1.0	
Male	156	1.2	(1.0–1.6)	1.2	(1.0–1.5)	140	0.8	(0.8–1.2)	1.1	(1.1–1.1)	214	1.2	(1.0–1.5)	1.2	(1.0–1.5)	415	1.1	(1.0–1.3)	1.1	(1.0–1.3)
Agegroup																				
18–49	19	1.0		1.0		9	1.0		1.0		19	1.0		1.0		17	1.0	1.0	1.0	
50–79	161	8.1	(5.1–13.1)	7.4	(4.6–12.1)	121	13.0	(6.6–25.5)	9.9	(5.0–19.7)	185	9.4	(5.9–15.1)	6.2	(3.8–10.1)	379	21.8	(13.4–35.4)	14.9	(9.1–24.3)
80-	110	16.8	(10.3–27.3)	15.2	(9.2–25.3)	164	53.8	(27.5–105.4)	37.1	(18.6–73.8)	197	30.8	(18.8–48.2)	18.0	(11.0–29.4)	412	74.6	(45.9–121.3)	44.8	(27.3–73.6)
Charlson comorbidity Index																				
0	116	1.0		1.0		78	1.0		1.0		95	1.0		1.0		194	1.0		1.0	
1	53	1.7	(1.2–2.3)	1.0	(0.7–1.4)	60	2.8	(2.0–4.0)	1.4	(1.0–1.9)	76	2.9	(2.2–4.0)	1.7	(1.2–2.3)	129	2.5	(2.0–3.1)	1.3	(1.0–1.6)
≥ 2	121	2.7	(2.1–3.5)	1.3	(1.0–1.7)	156	5.2	(4.0–6.9)	1.9	(1.4–2.5)	230	6.4	(5.0–8.1)	2.8	(2.2–3.7)	485	6.7	(5.7–8.0)	2.8	(2.3–3.3)
Arriving																				
Own accord	5	1.0		1.0		23	1.0		1.0		234	1.0		1.0		503	1.0		1.0	
Ambulance	174	111.6	(45.8–271.5)	71.4	(31.6–161.4)	182	25.7	(16.7–39.7)	18.5	(12.0–28.7)	128	1.8	(1.4–2.2)	1.7	(1.2–2.3)	277	1.8	(1.6–2.1)	1.2	(1.1–1.4)
MECU	111	1037.9	(422.3–2550.9)	781.6	(341.9–1786.8)	17	31.1	(16.5–58.4)	27.3	(14.5–51.3)	39	8.2	(5.8–11.6)	2.8	(2.2–3.7)	28	2.7	(1.8–3.9)	2.0	(1.4–3.0)
Day of arrival																				
Mon-Thursday	168	1.0		1.0		162	1.0		1.0		223	1.0		1.0		470	1.0		1.0	
Fri-Sunday	122	1.0	(0.8–1.3)	1.0	(0.8–1.3)	132	1.1	(0.9–1.4)	1.2	(0.9–1.5)	178	1.1	(0.9–1.4)	1.1	(0.9–1.4)	338	1.0	(0.9–1.1)	1.0	(0.9–1.2)
Time of arrival																				
8–15.59	143	1.0		1.0		158	1.0		1.0		204	1.0		1.0		441	1.0		1.0	
16–23.59	87	0.9	(0.7–1.1)	0.9	(0.7–1.2)	89	0.8	(0.6–1.0)	0.9	(0.7–1.1)	141	1.0	(0.8–1.2)	0.8	(0.8–1.3)	275	0.9	(0.8–1.0)	0.9	(0.8–1.1)
00–6.59	60	1.4	(1.0–1.9)	1.7	(1.2–2.6)	47	1.0	(0.7–1.4)	1.3	(0.9–1.8)	56	0.9	(0.7–1.2)	1.1	(0.8–1.5)	92	0.7	(0.6–0.9)	0.8	(0.7–1.1)
Urgency																				
Non urgent	9	1.0		1.0		13	1.0		1.0		39	1.0		1.0		141	1.0		1.0	
Urgent	74	3.7	(1.9–7.5)	3.1	(1.5–6.1)	132	4.6	(2.6–8.2)	3.7	(2.1–6.6)	244	2.9	(2.0–4.0	2.3	(1.7–3.3)	548	1.8	(1.5–2.1)	1.4	(1.2–1.7)
Very urgent	122	90.0	(45.6–177.4)	67.6	(34.2–133.5)	56	27.5	(15.0–50.4)	19.7	(10.7–36.2)	74	12.2	(8.3–18.1)	8.9	(6.0–13.1)	70	3.2	(2.5–4.5)	2.2	(1.6–3.0)
Missing	85	22.2	(11.2–44.2)	31.1	(15.6–61.9)	19	3.7	(1.8–7.5)	5.6	(2.8–11.2)	44	2.6	(1.7–4.0)	3.8	(2.5–5.9)	49	0.8	(0.6–1.1)	1.2	(0.8–1.6)
Vital parameters																				
Peripheral oxygen saturation (%)																				
≥ 90% +/− oxygen	125	1.0		1.0		190	1.0		1.0		293	1.0		1.0		640	1.0		1.0	
< 90% +/−oxygen	33	10.6	(7.2–15.7)	6.8	(4.6–10.1)	48	10.7	(7.7–14.8)	5.8	(4.2–8.1)	33	4.7	(3.2–6.7)	2.6	(1.8–3.7)	68	4.6	(3.5–5.9)	2.5	(1.9–3.3)
missing	132	4.0	(3.1–5.1)	5.7	(4.4–7.3)	56	1.1	(0.8–1.5)	1.7	(1.3–2.4)	75	1.0	(0.7–1.2)	1.4	(1.1–1.8)	100	0.6	(0.5–0.7)	0.9	(0.7–1.1)
Respiratory rate (rr/min)																				
5–36	134	1.0		1.0		212	1.0		1.0		306	1.0		1.0		660	1.0		1.0	
< 5	3	26.0	(7.8–86.7)	27.5	(7.9–95.7)	0	0	0	0	0	1	3.9	(0.5–28.9)	3.6	(0.5–28.0)	1	1.8	(0.2–13.2)	1.7	(0.2–12.8)
> 36	13	9.0	(5.0–16.1)	6.2	(3.5–11.2)	19	8.7	(5.4–14.1)	5.4	(3.3–8.9)	12	3.7	(2.1–6.7)	2.2	(1.2–4.0)	27	4.0	(2.7–6.0)	2.4	(1.6–3.7)
missing	140	2.9	(2.3–3.7)	4.2	(3.3–5.3)	63	0.8	(0.6–1.1)	1.2	(0.9–1.5)	82	0.7	(0.6–1.0)	1.1	(0.9–1.4)	120	0.5	(0.4–0.6)	0.8	(0.6–0.9)
Systolic Blood Pressure (mmHg)																				
≥ 90	170	1.0		1.0		235	1.0		1.0		343	1.0		1.0		728	1.0		1.0	
< 90	37	17.8	(12.3–26.7)	13.4	(9.2–19.4)	27	9.8	(6.5–14.8)	6.5	(4.2–9.8)	22	5.4	(3.5–8.4)	3.4	(2.2–5.3)	31	3.6	(2.5–5.3)	2.2	(1.5–3.3)
missing	83	2.7	(2.0–3.5)	4.3	(3.3–5.6)	32	0.7	(0.5–1.1)	1.4	(1.0–2.0)	36	0.6	(0.4–0.8)	0.9	(0.7–1.3)	49	0.4	(0.3–0.5)	0.6	(0.5–0.8)
Heart rate (hr/min)																				
40–140	190	1.0		1.0		245	1.0		1.0		352	1.0		1.0		742	1.0		1.0	
< 40	8	16.7	(8.0–34.8)	14.0	(6.6–29.8)	6	10.0	(4.3–23.0)	7.9	(3.3–19.0)	1	1.1	(0.2–7.9)	0.8	(0.1–5.7)	1	0.5	(0.1–3.7)	0.4	(0.0–2.6)
> 140	9	6.2	(3.1–12.2)	5.7	(2.9–11.4)	6	3.2	(1.4–7-4)	3.2	(1.4–7.3)	8	3.0	(1.5–6.2)	2.8	(1.4–5.8)	16	2.9	(1.8–4.9)	2.8	(1.6–4.7)
missing	83	2.4	(1.9–3.1)	3.9	(3.0–5.1)	37	0.8	0.6–1.2)	1.6	(1.1–2.2)	40	0.6	(0.5–0.9)	1.0	(0.8–1.)	49	0.4	(0.3–0.5)	0.6	(0.5–0.8)
Glasgow Coma Score																				
14–15	68	1.0		1.0		150	1.0		1.0		231	1.0		1.0		559	1.0		1.0	
3–13	100	53.5	(39.0–73.3)	45.2	(32.8–62.3)	45	11.3	(8.0–15.9)	7.8	(5.5–11.2)	44	7.1	(5.1–9.9)	4.9	(3.5–6.8)	55	3.7	(2.8–4.9)	2.5	(1.8–3.3)
missing	122	4.1	(3.0–5.5)	4.9	(3.6–6.6)	99	1.5	(1.2–1.9)	1.8	(1.4–2.4)	126	1.2	(1.0–1.5)	1.5	(1.2–1.8)	194	0.8	(0.7–0.9)	0.9	(0.8–1.1)
Temperature																				
35–39	108	1.0		1.0		191	1.0		1.0		279	1.0		1.0		641	1.0		1.0	
< 35	5	17.5	(7.0–43.9)	12.1	(4.8–30.8)	4	8.3	(3.0–22.8)	4.3	(5.3–11.8)	15	10.0	(4.6–21.8)	6.2	(2.8–13.8)	20	1.2	(0.3–4.7)	0.7	(0.2–2.7)
> 39	4	1.9	(0.7–5.1)	1.6	(0.6–4.4)	8	2.1	(1.0–4.3)	1.9	(0.9–3.8)	8	1.4	(0.7–2.9)	1.1	(0.6–2.3)	15	1.2	(0.7–1.9)	0.9	(0.6–1.6)
missing	173	4.9	(3.8–6.2)	6.1	(4.8–7.8)	91	1.4	(1.1–1.9)	1.9	(1.5–2.5)	99	1.0	(0.8–1.3)	1.3	(1.0–1.7)	132	0.6	(0.5–0.7)	0.8	(0.6–0.9)

^a^agegroup, gender, comorbidity

In contrast, impact of comorbidity increased with time from arrival to death and age and male sex were time stable risk factors. Fever and time and day of arrival showed no association with mortality in any of the groups.

Missing values were associated with immediate and 0–2 day mortality but had no association after 7 days (Table 2).

Discharge diagnoses from the ED showed that a majority of patients, who died immediately after arrival, were diagnosed with diseases of the circulatory system. This was also the major discharge diagnose-category in the other mortality groups, except for 8–30 day mortality, where diagnoses of the respiratory system accounted for the majority. Diseases of the respiratory system was the second largest group of ICD diagnoses in both the 0–2 day mortality and the 3–7 day mortality group. Injury, poisoning and external causes accounted for the second largest diagnose-category in the immediately dead group (Table 3).

**Table 3:** Frequency discharge diagnosis according to major ICD-10groups, distributed on four mortality levels

ICD-10 groups	Disease categories	Overall		0-4 h		4 h-2 day		3–7 day		8–30 day	
	Total N (%)	1721	(100)	290	(100)	222	(100)	N 401	(100)	N 808	(100)
A00-B99	Certain infectious and parasitic diseases	107	(6.2)	20	(6.9)	19	(8.6)	27	(6.7)	41	(5.1)
C00-D89	Neoplasms and diseases of blood and blodforming organs	153	(8.9)	5	(1.7)	12	(5.4)	33	(8.2)	103	(12.7)
E00-E90	Endocrine, nutritional and metabolic diseases	77	(4.5)	5	(1.7)	13	(5.9)	12	(3.0)	47	(5.8)
G00-G99	Diseases of the nervous system	17	(1.0)	2	(0.7)	0	0	8	(2.0)	7	(0.9)
I00-I99	Diseases of the circulatory system	454	(26.4)	159	(54.8)	82	(36.9)	103	(25.7)	110	(13.6)
J00-J99	Disease of the respiratory system	335	(19.5)	27	(9.3)	39	(17.6)	86	(21.4)	183	(22.6)
K00-K93	Diseases of the digestive system	161	(9.4)	11	(3.8)	25	(11.3)	43	(10.7)	82	(10.1)
N00-N99	Disease of the genitourinary system	54	(3.1)	2	(1.7)	5	(2.3)	10	(2.5)	37	(4.6)
R00-R99	Symptoms, signs, and abnormal clinical and labotory findings, not elsewhere classified	119	(6.9)	17	(5.9)	15	(6.8)	26	(6.5)	61	(7.5)
S00-T98	Injury, poisoning and certain other conseqeunces of external causes	91	(5.3)	30	(10.3)	2	(0.9)	21	(5.2)	38	(4.7)
	Others (mental, eye, ear, skin, connective tissue, external causes etc)	147	(8.5)	10	(3.4)	9	(7.7)	31	(7.7)	97	(12.0)
	Missing	6	(0.3)	2	(0.7)	1	(0.5)	1	(0.2)	2	(0.2)

## Discussion

We showed an over-all-30 day mortality of 4%. One third of the 30 day mortality occurred within 2 days after arrival to the ED. Abnormal vital signs are strong risk factors for mortality within the first 2 days and hereafter the association declines with time from arrival.

Our overall mortality is similar to previous results from other ED-settings [12, 23]. Also, abnormal vital parameters and the degree of acuity at arrival are also well-known risk factors [3, 4].

Timing related to mortality as outcome is often not taken into consideration, when weighing the effect of risk factors. In our study, abnormal vital parameters and degree of acuity at admission are strongly associated with mortality in the first hours and days after admission, where after the association decreases. The effect of other risk factors such as male gender, comorbidity and high age are time stable or even increasing over time.

We excluded patients arriving at own accord with minor trauma, since these patients are only registered with a minimum of data and often not initially triaged. By doing this, we excluded a fraction of clinically stabile orthopedic patients, who were admitted directly in the orthopedic department for observation or surgery or discharged directly after examination. Many could have been assessed by a physician in the primary sector.

Patients who were not registered dead by a physician prior to ED arrival were included. Patients with major trauma or other seriously life-threatening clinical conditions, who were kept alive by active treatment from the medical staff at MECU, during transfer to the ED, are included. In cases, where active treatment ceased shortly after arrival to the ED and the patient died, they were included in the immediately dead group and add strength to the association with mortality in all parameters. In reality, active treatment is often ceased due to detailed knowledge on severe comorbidity, initial trauma scans or lab results which makes further treatment unethical.

A very large proportion of vital parameters and registration of urgency level were missing, particularly in the group of patients dying 0–4 h after arrival. This supports the well-known finding, that large amounts of missing data are present in trauma or acute-setting registries and is also a consequence of the use of secondary data sources where selection, quality and method of collection is not under the control of the researcher [24–26]. In our study, missing values in all variables are associated with 0–2 day mortality, but the association disappears over time and missing data show no association after 7 days.

Our result reasserts the interpretation that registration and documentation in patient records is not always carried out due to logistics, prioritization, investigations and treatment in very acute situations, with very ill patients. One might also conclude that it can be fatal to miss vital parameter measurement and triage upon arrival to the ED if the patient is very sick, because lacking recognition of a serious clinical condition at arrival, can lead to dead within the first few days. Finally, the patient might be in such poor clinical condition at arrival, that the healthcare personnel can acknowledge the severity without measuring vital values.

The occurrence of missing data is a well-known premise of data from acute settings and in our studies, missing data in vital values and triage might represent immeasurable low or high values, values of zero, normal values observed but not registered, or simple lack of registration due to circumstances surrounding the patient at the time of assessment. The results in the multivariate analysis of this article reflect the fact that missing values are not missing at random, as OR related to missing data was high. If we were to omit these data, important information and future research aspects of risk factors associated with mortality in the ED would be lost. We do agree that missing data is a limitation of the study and we acknowledge that results in our multivariate analysis suffer to these missing data. In the multivariate analysis we have handled missing values as independent variable. As OR related to missing values are very high, this result in a conservative OR estimate for patients with registered abnormal vital values closer to one than we believe it would have been if no data were missing.

We have not taken do-not-attempt-to-resuscitate orders or terminal diagnoses into account. These patients are often admitted to palliative care until death or discharged to home after initial assessment. They are also a group of patients with many missing data in vital values. However, these patients are always initially triaged including registration of vital values upon arrival to the ED and therefore included in our analysis.

The impact of risk factors does not only vary according to population but also vary over time.

In the overall context, the results concerning risk factors associated with short term mortality following acute ED-admission are inconsistent, and studies differ in population, selection and outcome mortality timespans. Many studies concerning adverse events in the ED are focused on logistic and environmental factors and clinical condition at arrival. The distribution and differences of OR across mortality groups in our study, show that interpretation of studies conducted in emergency settings, should take outcome mortality measures into account, when comparing studies on risk factors and developing risk prognostication models.

### Strength and limitations

The main strength of our study is the cohort, consisting of consecutive patients registered in the ED during the study period. All patients had complete follow-up data, combined with data from national, validated registries, which are highly complete. The present single-center study focused on an ED at a University Hospital and interpretations may not be generalizable to other settings. However, our study cohort comprised of broad typical ED population, with patients entering the ED, due to medical or surgical conditions. Thus, our results reflect a highly diverse patient group.

In theory, all ED visits in Denmark are preceded by contact with a physician from primary sector, thereby avoiding unnecessary contacts to the ED. The proportion of events are, however, probably similar to other ED’s with similar logistic and regional setup, since patients eventually admitted are probably alike [27].

We chose to use ED-discharge-diagnoses and not hospital-discharge-diagnoses, since complications to the primary condition might later occur and cause registration of different discharge diagnoses.

Missing data is always a limitation to a study and the proportion of missing in databases of acute settings are well-known. Several research groups have evaluated the effects of missing data on analysis from trauma databases and different techniques for handling missing data [28, 29]. In our study, missing data in vital values may represent immeasurable low or high values, normal values observed but not registered, values of zero or simple lack of registration due to other circumstances surrounding the patient. We have chosen to handle missing data as an independent variable in the multivariable analysis and find that the association with mortality demonstrates that missing data is not missing at random.

The reporting of this study conforms to the STROBE statement [30].

## Conclusion

Among adult patients registered alive at arrival in the ED, we showed an over-all 30-day mortality of 4%. Approximately one third dies within 2 days after arrival to the ED. Strong risk factors for immediate mortality are abnormal vital values and a high degree of acuity at arrival to the ED. These risk factors declined considerable according to increasing time from arrival till death. In contrast increasing age, male sex and increasing comorbidity were all mortality risk factors, but their odds ratios did not diminish according to time from arrival to death (Tables 2 and 3).

## Additional file

Additional file 1: DEPT triage. Vital values. (XLSX 61.8 kb)

## Abbreviations

95%CI: 95% confidence interval
ED: Emergency Department
MECU: Mobile Emergency Care Unit
OR: Odds ratio
OUH: Odense University Hospital
